# Precision Vascular Delivery of Agrochemicals with Micromilled Microneedles (µMMNs)

**DOI:** 10.1038/s41598-019-50386-8

**Published:** 2019-09-30

**Authors:** Avra Kundu, Maria Gabriela Nogueira Campos, Swadeshmukul Santra, Swaminathan Rajaraman

**Affiliations:** 1 NanoScience Technology Center (NSTC), University of Central Florida, Orlando, FL 32826 USA; 2 Department of Materials Science & Engineering, University of Central Florida, Orlando, FL 32816 USA; 3 Department of Electrical & Computer Engineering, University of Central Florida, Orlando, FL 32816 USA; 4 Department of Chemistry, University of Central Florida, Orlando, FL 32816 USA; 5 Burnett School of Biomedical Sciences, University of Central Florida, Orlando, FL 32827 USA

**Keywords:** Mass spectrometry, Microengraving

## Abstract

We demonstrate use of makerspace techniques involving subtractive microtechnologies to fabricate micromilled microneedles (µMMNs) of stainless steel (SS) for precise delivery of agrochemicals into vascular bundles of plant tissue. Precision delivery is of immense importance for systemic pathogen control in specific areas of plant tissue. Optimization of the micromilling allows for selective removal of SS at the microscale and the microfabrication of a 5 × 5 array of µMMNs having both base width and height of 500 µm to enable precise puncture into the stem of citrus saplings. Atomic Absorption Spectroscopy reveals up to 7.5× increase in the uptake of a therapeutic cargo while Scanning Electron Microscopy reveals that specific sites of the vascular bundle; either xylem or the phloem can be uniquely targeted with customized µMMNs. Such rapid and cost-effective customization with intricate designs along with scalability is enabled by makerspace microfabrication. Additionally, a 19 × 20 array of micromilled *mesoneedles* has been fabricated and affixed to a paint roller as an applicator system for real-world field testing outside the laboratory. Initial results indicate reliable behavior of the applicator system and the technique can be applied to the systemic delivery of agrochemicals while conserving the loss of the agrochemical with increased application efficiency.

## Introduction

Food production is one of the main challenges to be overcome in the future. By 2050, world population is expected to reach 9 billion inhabitants and food production must be doubled in the next 30 years^1^. Efficient delivery of agrochemicals, such as fertilizers and pesticides, is one of the key elements for improved food productivity and security. Current developments in this area include fertigation, in-field sensors for automatized sprays based on plant demand, use of drones, etc.^2^. Nevertheless, smart agriculture technologies are expensive and require large investments. Another challenge on efficient delivery of agrochemicals is related to systemic delivery throughout the plant. Foliar spray^3^ and soil drench^4^ are the standard methods to apply agrochemicals such as pesticides and fertilizers. However, these application methods have some negative environmental implications such agrochemical run-off and adverse effect on the soil microbiome. Furthermore only a small percentage of applied agrochemical is actually up taken by the plant^5^.

Scientific reports suggest that plants are capable of sensing their environment in some way similar to humans and other animals^6^. Recent discoveries indicate that the very root apex of a plant has the capacity to detect twenty (20) different physical and chemical parameters including gravity, light, magnetic field, pathogens and more^6^. Therefore, devices and mechanisms used to treat infections and diseases in humans can potentially be translated for treatment of plants as well. Microneedle (MN) based treatment of plants infected with pathogens is one such overlapping application arena. MNs were first envisioned as painless drug delivery devices decades ago, but with the accelerated evolution of microfabrication techniques, MNs have transitioned from academic laboratories to pharmaceutical companies as commercially available, off-the-shelf products^7,8^. Hollow and solid microneedle technologies are commonly used in drug delivery systems^7^. MNs offer patient-friendly delivery solutions for vaccines or difficult-to-deliver biologics particularly for hypodermic needle-phobic patients^8^. Such transdermal MNs work by creating micron sized pores in the skin to enhance delivery of the drug across the skin without stimulating the pain nerves^8^ demonstrating the immense degree of control that can be obtained with dermal penetration utilizing properly designed MNs. Such controlled penetration to specific depths is of special importance for agricultural applications as systemic pathogens in plants reside in hard to reach areas of the plant tissue^9^. As examples, bacterium such as *Candidatus* Liberibacter asiaticus, responsible for Huanglongbing (HLB, also known as citrus greening)^10^ reside in the phloem tissue of the plant while *Xylella fastidiosa*, responsible for citrus variegated chlorosis reside in the xylem tissue^11^. As a result, custom fabricated MNs would allow for disease and site-specific vascular treatment of plants using agrochemicals. Additionally, they can address concerns relating to the systemic delivery of agrochemicals while conserving the loss of the applied agrochemical with increased rainfastness.

In recent years there has been a gradual transformation in the micromachining of biological microdevices such as MNs^12^. Traditional cleanroom-based microfabrication approaches are being replaced by non-conventional techniques outside the cleanroom which allows for the use of a different tool-set while offering a much larger material palette along with rapid fabrication timeframes, design modifications on-the-fly, cost effective, and scalable fabrication. The authors have previously introduced the concept of and demonstrated the use of ‘Makerspace Microfabrication’^13,14^ for fabricating MNs deployed in transdermal drug delivery applications. These MNs were fabricated using micro-stereo lithography (µSLA), an additive manufacturing technique^13^. Such MNs are appropriate for penetrating soft tissue like skin having an Ultimate Tensile Strength (UTS) of ~40 MPa^13^ since commercially available 3D printed materials can have an UTS only as high as 65 MPa^15,16^. However, for penetrating trees, the UTS of the material used in MN fabrication needs to be an order of magnitude higher (~500 MPa^17^), making a material such as stainless steel (SS) with similar tensile strengths (UTS of ~500 MPa^18^), a much better choice. SS based MNs can be fabricated using micromilling which is a subtractive manufacturing method^19,20^ that creates microscale features utilizing microscale cutting tools to remove unwanted bulk material to define the desired geometry. The microscale cutting tools vary in diameter from 5 to 400μm and have edge radius that varies from 1 to 10 μm^21^.

In this paper, we demonstrate the use of micromilled microneedles (µMMNs) for vascular delivery of agrochemicals to highly specific and targeted sites of the plant tissue. With optimized micromilling conditions, a µMMN array is fabricated in a 5 × 5 configuration on a planar stainless steel sheet having a thickness of ~100 µm in the traditional “Washington Monument” design. The microneedles are subsequently transitioned into the third dimension using a customized Hypo-Rig^22^ to have the 3D µMMN array. The microneedles have a base width of ~500 µm and a height of ~500 µm. These minimally invasive stainless steel microneedles were used to create controlled and targeted punctures in the stem of citrus saplings and a model therapeutic cargo of a zinc-based antimicrobial called Zinkicide™^23^ was delivered and the study of the uptake mechanism into the leaves, stem and roots was performed. Atomic Absorption Spectroscopy (AAS) reveals that the saplings punctured with µMMNs and treated with the model therapeutic showed significant increase in the cargo uptake in the leaves (6×) and the stem (7.5×) when compared to the control sample. Scanning Electron Microscopy (SEM) imaging reveals that the µMMNs are capable of making targeted site-specific punctures to the xylem and the phloem regions. The micromilling process has been extended to demonstrate its ability to realize intricate geometries such as “Tridents” and “Triangular Tip” toward applications to fully grown trees. Both a 6 × 6 array of “trident” shaped *mesoneedles* having a height of 4 mm and a 19 × 20 array of “triangular tip” *mesoneedles* with similar dimensions are demonstrated in this work. Owing to the flexible nature of the ~100 µm SS substrate, this design was affixed on a roller and tested in field conditions to demonstrate scalability and real-world deployment of our methods. This precision transport of agrochemicals to specific target sites provides a low-cost alternative to delivery of agrochemicals through the stem, directly to the plant tissue, without impacting soil beneficial microorganisms and with no risk of rainfastness.

**Figure 1:**
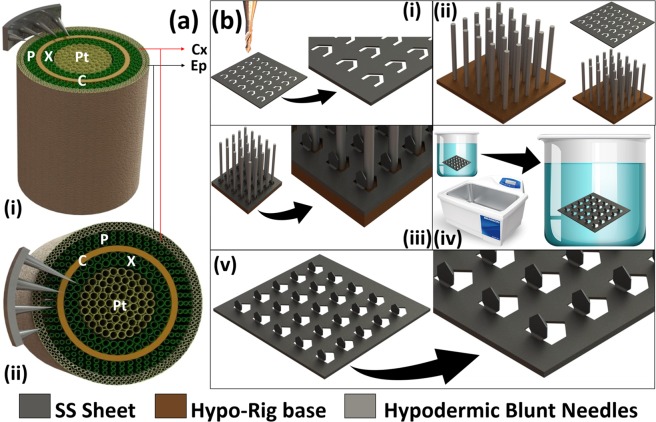
(**a**) Concept schematic on the usage of µMMNs for controlled penetration in plant stem tissue. (i) Isometric view and (ii) Top view. (**b**) Fabrication technique used to realize the µMMNs using ‘Makerspace Microfabrication’. (i) Micromilling onto planar stainless steel substrate (SS); (ii) Aligning the SS substrate with the Hypo-Rig; (iii) Transitioning the µMMNs out of plane; (iv) Acid pickling with sonication to remove debris from micromilling and (v) Final µMMNs ready for testing.

Schematic of the concept of µMMNs for controlled, site-specific penetration in plant stem tissue is shown in Fig. 1(a): (i) and (ii). It is proposed that tuning of the MN geometry for controlled puncture of specific target sapling/tree sites such as the xylem and phloem is possible. Figure 1(b):(i–v)) depict the fabrication technique used to realize the µMMNs using ‘Makerspace Microfabrication’. The micromilling tool is used to engrave the “Washington Monument” design onto a planar SS substrate [Fig. 1(b:i)]. The engraving entails selective removal of the SS material. A custom fabricated Hypo-Rig is used to transition the “Washington Monument” structures out-of-plane resulting in the formation of 3D µMMNs [Fig. 1(b):(ii) and (iii)]. Acid pickling^24^ of the µMMNs allows for not only the removal of unwanted debris from the micromilling process but also potential sharpening of the µMMN tips [Fig. 1(b):(iv)] to construct final µMMNs ready for testing [Fig. 1(b):(v)].

## Results and Discussion

**Figure 2:**
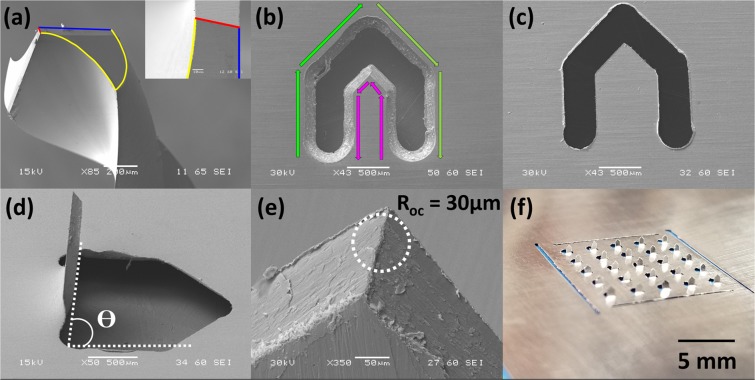
(**a**) SEM image of the micromilling pointed tool; (**b**) top side of the SS sheet after micromilling; (**c**) bottom side of the micromilled SS sheet; (**d**) SEM of the near perpendicular alignment of a single µMMN to the horizontal; (**e**) Tip of a single µMMN depicting a radius of curvature of 30 µm and (**f**) Photomicrograph of the 5 × 5 array of µMMNs.

Figure 2(a) shows the SEM image of the micromilling pointed tool used to engrave patterns onto the SS substrate. As observed in the image, the micromilling tool has a web thickness (marked in red) of 40 µm^25^. The two (2) cutting lips (or flutes) of the tool and the landing of the tool are highlighted in blue and yellow respectively. The number of flutes is one of the governing factors while calculating the chip load on the tool. With a feed rate of 3 mm/s and a spindle frequency of 1000 Hz, the chip load on the tool is ~1.5 µm as Chip load = (Feed rate)/(Frequency × Number of flutes)^26^. The values of the spindle frequency and feed rate were chosen so that the chip load would remain under 10% of the minimum tool dimension (40 µm web thickness in this case). A close-up SEM of the pointed tip is also shown in the inset of the same figure. Figure 2(b) depicts the top side of a micromilled SS sheet. It is observed that the micromilling tool indents a characteristic 45° slant face on the top side as the micromilling tool has a 90° pointed tip with respect to the SS surface. As the milling tool rotates in a clockwise direction and moves with respect to the substrate while etching the desired geometry, the edges of the entire needle (eventually the 3D geometry) are defined by the down-milling (highlighted in magenta in Fig. 2(b)) while the in-plane geometry is defined by up-milling (highlighted in green in Fig. 2(b))^27^. An optimized feed-rate of 3 mm/s allows for both the edges to be smooth as observed in the figure. Higher feed rates would translate to a greater chip load which leads to more wear and eventually breakage of the tool while lower feed rates would lead to poor device finish, inefficient cycle times, and premature tool wear due to increased tool indentation as a result of chip thinning^28^. Some debris from the micromilling process can adhere to the substrate as also shown in the figure but this material is removed by an acid pickling process^24^. Figure 2(c) shows the bottom side of the micromilled SS sheet which only shows the cut-out of the “Washington Monument” design and not the milled features as this side was not exposed to the milling tool. Figure 2(d) depicts the µMMN after the planar micromilled cut-out has been transitioned out of plane. The µMMN have a very tight angular distribution (ϴ) of 85.2° [Fig. S1(a)] with the horizontal which shows the efficiency of the Hypo-Rig for transitioning the µMMNs out-of-plane as observed in Fig. 2(d,f). The transition process proceeds from the bottom face of the SS sheet which naturally lends the µMMNs to have a sharp slicing tip as observed in Fig. 2(e). The radius of curvature (R_OC_) of the slicing tip is found to be ~30 µm. Figure 2(f) shows the optical photomicrograph of the µMMNs fabricated in a 5 × 5 configuration. The microneedles were designed to have a base width of ~500 µm and a height of ~500 µm. It is observed that the µMMN dimension closely matches the design dimension. A box plot of N = 25 µMMNs (one µMMN patch) showing variation in height and base width is shown in Fig. S1(b,c). A mean height and width of 550.6 µm and 466.8 µm is obtained for the µMMNs respectively. A standard deviation of 42.57 µm and 31.83 µm from the design values (both less than 10%) is obtained for the height and base width respectively due to the micromilling process.

**Figure 3:**
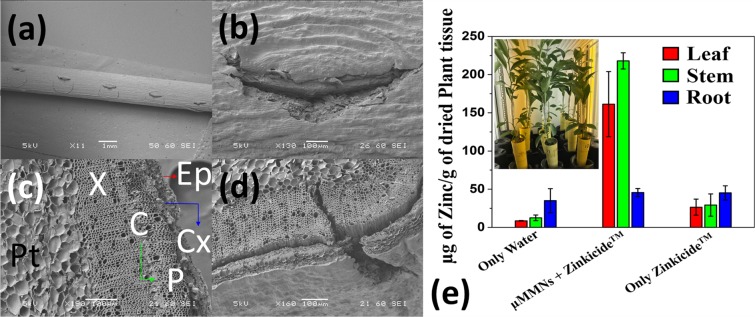
(**a**) SEM image (5 × 1 penetrations) of the puncture caused by the µMMN onto the stem of a citrus seedlings with (**b**) showing a close-up SEM image of one puncture site; (**c**) SEM image showing the cross-section of the un-punctured stem with the epidermis (Ep), cortex (Cx), phloem (P), cambium (C), xylem (X) and the pith (Pt); (**d**) SEM image of the cross-section of a stem at one of the µMMN puncture sites; (**e**) Bar graph of the of the Zn concentration in the leaves, stem and roots after the application of the therapeutic cargo of Zinkicide™. Inset shows the plants in the growth chamber, after puncturing with the µMMN and sealing the plastic container containing the therapeutic cargo around the puncture site.

Figure 3(a) shows a SEM image of the puncture caused by a µMMN onto the stem of the citrus seedlings. An array of one entire row is (5 × 1) is clearly observed in the image and two such punctures were made on either side of the stem for delivery of the therapeutic cargo. Having a µMMN patch in a 5 × 5 configuration allows it to be used with saplings in various stages of growth. As the sapling matures, the stem increases in diameter and the entire patch would conform onto the surface of the stem. In this work we used saplings that are 12 months-old and had a maximum stem diameter of 2.5 mm. Therefore, penetrations of two entire rows (5 × 1) on either side of the sapling stem (10 penetrations in total) was attempted to study the effect of the therapeutic cargo. The design featuring a patch in a 5 × 5 configuration provides for redundancy in case of puncture failures. A box plot of N = 10 puncture sites showing variation in puncture width is depicted in Fig. S1(d). It is observed that the width of the puncture is 683 µm with a standard deviation of 35.22 µm. The larger width of the puncture site is attributed to the shear forces during the puncture of the stem with the µMMNs. Figure 3(b) shows the close-up SEM image of one puncture site caused by a single µMMN. The sharpness of the µMMNs causes stem penetration with minimal damage to neighboring tissue. This would allow for the stem to heal rapidly post-treatment resulting in reduced secondary infections from the wounds caused by the µMMN.

Figure 3(c) shows the enlarged cross-section of the un-punctured stem with the epidermis (Ep), cortex (Cx), phloem (P), cambium (C), xylem (X) and the pith (Pt) highlighted in the figure^29^. A lower magnification image of the cross-section of the un-punctured stem is shown in Fig. S2. The seedlings used in our experiments were 12 months old and the resulting the vascular tissue are in their developing stages and are therefore not very distinct but can clearly be differentiated under the SEM. Figure 3(d) shows a cross-sectional SEM of the stem at one of the µMMN puncture sites. The µMMN is clearly observed to puncture the Ep and Cx layers and create a pathway through the vascular tissue. Figure 3(e) depicts the bar graph of the AAS results quantifying the Zn concentration in the leaves, stem and roots after the application of the therapeutic cargo consisting of Zinkicide™. The inset shows the plants in the growth chamber, after puncturing with the µMMNs and sealing the plastic container containing the therapeutic cargo around the puncture site. The zinc content in stem and leaves after microneedle treatment is observed to have increased 7.5× and 6× respectively with respect to the control. These results suggest the transport of zinc through the xylem tissue. As the xylem allows for unidirectional transport (upward), the increase in zinc concentration in the leaves and stem and not the roots portrays µMMN penetration into the xylem region. This is also corroborated with the SEM images [Fig. 3(d)]. No significant increase of zinc content in the roots after treatment with Zinkicide™ indicates that the µMMN did not selectively penetrate the phloem tissue, which is multidirectional and would have allowed for zinc transport to the roots as well. However, with custom design of the µMMNs, accounting for the thickness of the stem and depth of the phloem tissue, micromilling-based rapid realization of MNs would make it possible to selectively deliver to the phloem region. This would be of significant importance as it would be an effective means of treating Huanglongbing (HLB, also known as citrus greening) which is a systemic bacterial disease caused by *C*andidatus Liberibacter asiaticus (*C*Las) which requires bactericides (including currently used antibiotics) to be delivered directly to the phloem of the plants.

**Figure 4:**
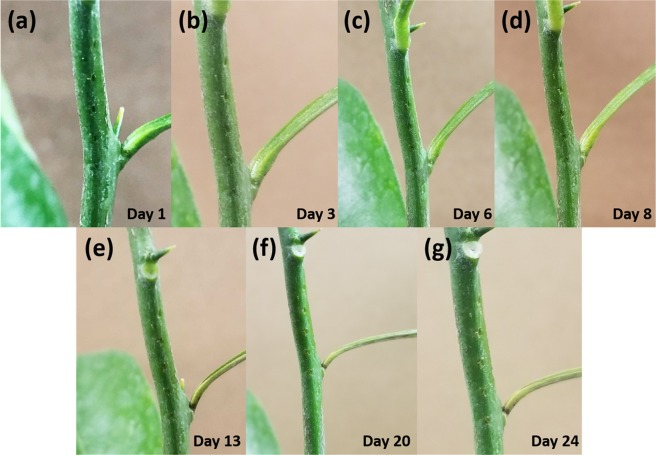
(**a**–**g**) Self-healing of the puncture sites caused by the µMMNs from Day 1 (day of puncture), Day 3 (healing of wound) and Day 24 (scar healing).

Figure 4(a–g) shows the self-healing of the puncture sites caused by the µMMNs. As evident from the figure, the puncture sites start self-healing in a few days, with visible scars from the wound starting to close up. Based on the visual observation, most wounds disappear by Day 24 even though the outline of the scar is noticeable. Figure S3(a) shows the box plot of N = 10 µMMNs post puncture sites onto the citrus stem after M = 6 cycles. Each cycle corresponds to the penetration of the two 5 × 1 arrays onto the citrus stem. It is interesting to note here that the µMMNs do not break after repeated, successful citrus stem penetration affirming the hypothesis that the large Young’s Modulus of SS can overcome the UTS of citrus stems. However, as observed in the figure, if the µMMNs are not correctly aligned to the plant stem, they may be bent at other angles after treatment. Although the flexible nature of the SS substrate allows for the µMMNs to conform to the curvature of citrus stem, obtuse or acute angles can result due to improper alignment as observed in Fig. S3(b,c) respectively. Nonetheless, a majority of the µMMNs maintain fabricated angular distribution and as a result the array can be reused multiple times.

**Figure 5:**
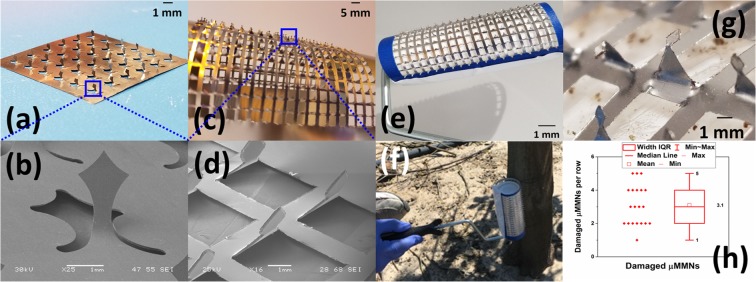
(**a**) Photomicrograph of an array of 6 × 6 trident shaped *mesoneedles* with (**b**) SEM image showing the intricate design features; (**c**) Photomicrograph of an array of 19 × 20 *mesoneedles* with a triangular tip with the (**d**) SEM image of the *mesoneedle* showing the triangular tip; (**e**) Photomicrograph of a 19 × 20 *mesoneedle* array affixed onto a paint roller with (**f**) field testing of the applicator system; (**g**) Optical micrograph of damaged needles in the paint roller based applicator system after rolling onto the tree and (**h**) number of *mesoneedles* which were bent after N = 5 rolling operations performed by the applicator system.

To demonstrate scalable micro-manufacturing and field-readiness of the µMMNs in a real-world setting outside the lab, two distinct aspects of the reported technology are highlighted and demonstrated. First, the versatility of the micromilling process to realize any customized design and second preliminary results of a roller array based applicator system used on a citrus tree are demonstrated in this paper. Figure 5(a) shows a photomicrograph of an array of 6 × 6 trident shaped *mesoneedles* having a height of ~4 mm suitable for use on fully grown trees. Figure 5(b) shows the intricate details of the “Trident” in the microscale achieved using micromilling allowing for meso to micro-scale precision of the micromilling technology. Unlike the “Washington Monument” design, the “Trident” design may also allow for extraction of plant tissue with the two pointed edges on either side during withdrawal of the needles allowing for potential plant histology and will allow for better anchoring during penetration. Figure 5(c) shows an array of 19 × 20 *mesoneedles* with a “Triangular tip” design with the microscale features highlighted in the SEM image of the *mesoneedle* in Fig. 5(d). As the ~100 µm SS substrate is flexible as seen in Fig. 5(c), it can conformally attach to practical applicator systems. The 19 × 20 array which spans a total area of (95 × 110) mm^2^ is affixed onto a commercial paint roller applicator system [Fig. 5(e)] and applied to the bark of a citrus tree parallel to the ground surface as observed in Fig. 5(f). This horizontal approach of rolling the applicator ensures that an entire row of needles (N = 19) on the roller engage completely with the cylindrical trunk of the tree. As the axis of the two cylinders, namely the tree trunk and the paint roller applicator are parallel to one another the applicator can roll over the cylindrical conformity of the tree trunk. In this case, an entire row of needles on the roller make full contact with the bark of the tree. This ensures (a) sufficient punctures on the bark of the tree in one rolling action and (b) the entire force applied during the rolling action being transferred to the tips of the needles in one entire row and does not lead to premature roller failure in specific sections of the device. A schematic illustrating the difference between the horizontal approach and vertical approach of the applicator is provided in Fig. S4.

The microneedle array successfully penetrated the tree in the regions where the microneedle loaded applicator system was delivered. To assess the reliability of the µMMN applicator system in the demonstrated “in field application”, a failure analysis of the microneedles was carried out to study the efficacy of the system. As already discussed earlier, alignment of the applicator to the tree bark has to be accounted for since all MNs do not align in such a system. These needles were marked as damaged as observed in Fig. 5(g). Figure 5(h) shows the number of *mesoneedles* which were bent in non-vertical angles after N = 5 applications using the system. It is observed that the total number of needles which were bent due to five successive applications was ~15% demonstrating an average of three (3) mesoneedles per row.

## Conclusions

Micromilled microneedles (µMMNs) for precision vascular delivery of agrochemicals has been successfully demonstrated. It is seen that makerspace enabled microfabrication allows for rapid, robust, benchtop based, cost-effective fabrication for realization of micro and mesoscale needles which can target specific portions of the vascular bundle of plants, for example xylem and the phloem. µMMNs are able to penetrate plant tissue in a minimally invasive fashion enabling rapid self-healing. A 7.5× increase in the uptake of a therapeutic cargo of Zinkicide™ shows the effectiveness of the puncture and vascular delivery mechanisms. Further, the methodology is capable of rapid and cost-effective customization and has been demonstrated to be scalable and field-ready with an array of 19 × 20 *mesoneedles* having reliable behavior in real world settings.

## Methods

### µMMN fabrication

For the fabrication of the 5 × 5 MN array with “Washington Monument” design (chip size: 17 mm × 17 mm), a 90-degree T-4 Mill Tool (T- Tech, Peachtree Corners, GA, USA) was spun at 60,000 rpm (1000 Hz) in a T-Tech QC-J5 Quick Circuit Prototyping Systems to cut into a stainless steel sheet (~100 µm thick; Trinity Brand Industries, Countryside, IL, USA). The feed rate was maintained at 3 mm/sec with a depth of cut ≥100 µm. A custom Hypo-Rig was used to transition the µMMNs out of plane. This technology is essentially a custom designed array of hypodermic needles having the exact pitch and number of the MNs that need to be transitioned out-of-plane. The entire array is housed on a custom designed jig with matching dimensions and assembled using proprietary techniques. The 3D µMMNs were subsequently pickled in a solution of DI Water (80 wt%): 70% HNO_3_ (11 wt%): 49% HF (9 wt%) at 50 °C for 3 minutes with sonication. The 6 × 6 “Trident” (chip size: 40 mm × 40 mm) and 19 × 20 (chip size: 95 mm × 110 mm) “Triangular” MN arrays were micromilled with the same parameters using the appropriate CAD design. For the µMMN roller, the ~100 µm SS sheet with the µMMN array was affixed onto a paint roller frame with adhesive tape after removing the fabric on the paint roller.

### µMMN puncture onto saplings and trees

Citrus seedlings (Citrus reshini, Cleopatra mandarin) approximately 12 month-old were used as plant model for the experiments (6 plants per group). The stem area (about 10 cm above the soil) was indented with µMMNs and covered with plastic container containing 2 mL of each of the therapeutic cargo (5000 ppm Zinkicide™) or Deionized (DI) water. The stem area of the microneedle control group of plant was only covered with the treatments, without being indented by the µMMNs. Plants were kept in a growth chamber (Panasonic Environmental Test Chamber, MLR-352H, Japan) for 48 hours. Controlled day/night cycling temperature, light intensity and humidity were used to simulate the weather conditions of Florida during summer (temperature >26.67 °C with a relative humidity of 60–80%). For the *mesoneedle* array applicator system affixed onto a paint roller, a six-year-old ‘Ruby Red’ grapefruit tree located in the Estes Citrus Inc. grove at Vero Beach, Indian River County, Florida was treated in March 14^th^, 2018. The trunk was rolled about 15 cm above the soil level. The rolling direction was parallel to the ground level.

### Atomic absorption spectroscopy

The plants were taken out from the growth chamber and the plastic container was detached and plants were removed from soil. They were washed and separated in parts (roots, leaves and stem) before being dried in an oven at 45 °C for 24 hours. Dry parts were weighed, ground and digested with nitric acid, hydrochloric acid and hydrogen peroxide (EPA recommended methodology)^30^ for Zn content analysis by Atomic Absorption Spectroscopy using Perkin Elmer Analyst 400 Atomic Absorption Spectrometer (Perkin Elmer, MA, USA). The results were plotted by microgram (µg) of metallic Zn per gram (g) of dried plant material.

### SEM imaging

Scanning electron microscope (SEM) imaging of the µMMN array was performed using JSM 6480 (JEOL, Peabody, MA, USA). For plant tissue imaging, lyophilization of the samples was performed. To obtain a slow freezing rate, the stems were frozen at −12 °C for 24 hours. To prevent the samples from being disturbed when vacuum was introduced, the stems were placed in a freeze-drying container and covered with a polystyrene petri-dish with drilled holes to allow vapor to escape. The container was subsequently connected to a sample valve on the drying chamber of a 1 liter benchtop freeze-dry system (FreeZone, Labconco, Kansas City, Missouri). The samples were dried for 12 hours at a vacuum level of 0.033 mbar with a collector temperature of −40 °C. After 12 hours, the dried samples were removed and ready to be imaged.

## Supplementary information

Supplementary Information

## Data Availability

All data generated or analyzed during this study are included in this published article (and its Supplementary Information files).
