# Supplementary material for: Precision Vascular Delivery of Agrochemicals with Micromilled Microneedles (µMMNs)
Source: Sci Rep. 2019 Sep 30;9:14008. doi: 10.1038/s41598-019-50386-8 (PMC6768873; doi:10.1038/s41598-019-50386-8)
Supplement: Supplementary file 1 — Supplementary Information [file 41598_2019_50386_MOESM1_ESM.docx]

**SUPPLEMENTARY INFORMATION**

**Precision Vascular Delivery of Agrochemicals with Micromilled Microneedles (µMMNs)**

*Avra Kundu^1^, Maria Gabriela Nogueira Campos^1^, Swadeshmukul Santra^1, 2, 4, 5^ and Swaminathan Rajaraman^1, 2, 3, 5*^*

^1^NanoScience Technology Center (NSTC), University of Central Florida, Orlando, FL 32826, USA.

^2^Department of Material Science & Engineering, University of Central Florida, Orlando, FL 32826, USA.

^3^Department of Electrical & Computer Engineering, University of Central Florida, Orlando, FL 32826, USA.

^4^Department of Chemistry, University of Central Florida, Orlando, FL 32816, USA.

^5^Burnett School of Biomedical Sciences, University of Central Florida, Orlando, FL 32827, USA.

Prof. Swaminathan Rajaraman

[*Swaminathan.Rajaraman@ucf.edu](mailto:*Swaminathan.Rajaraman@ucf.edu); Tel.: +1-407-823-4339

Research 1, Office 237

4353 Scorpius Street

Orlando, FL 32816-0120


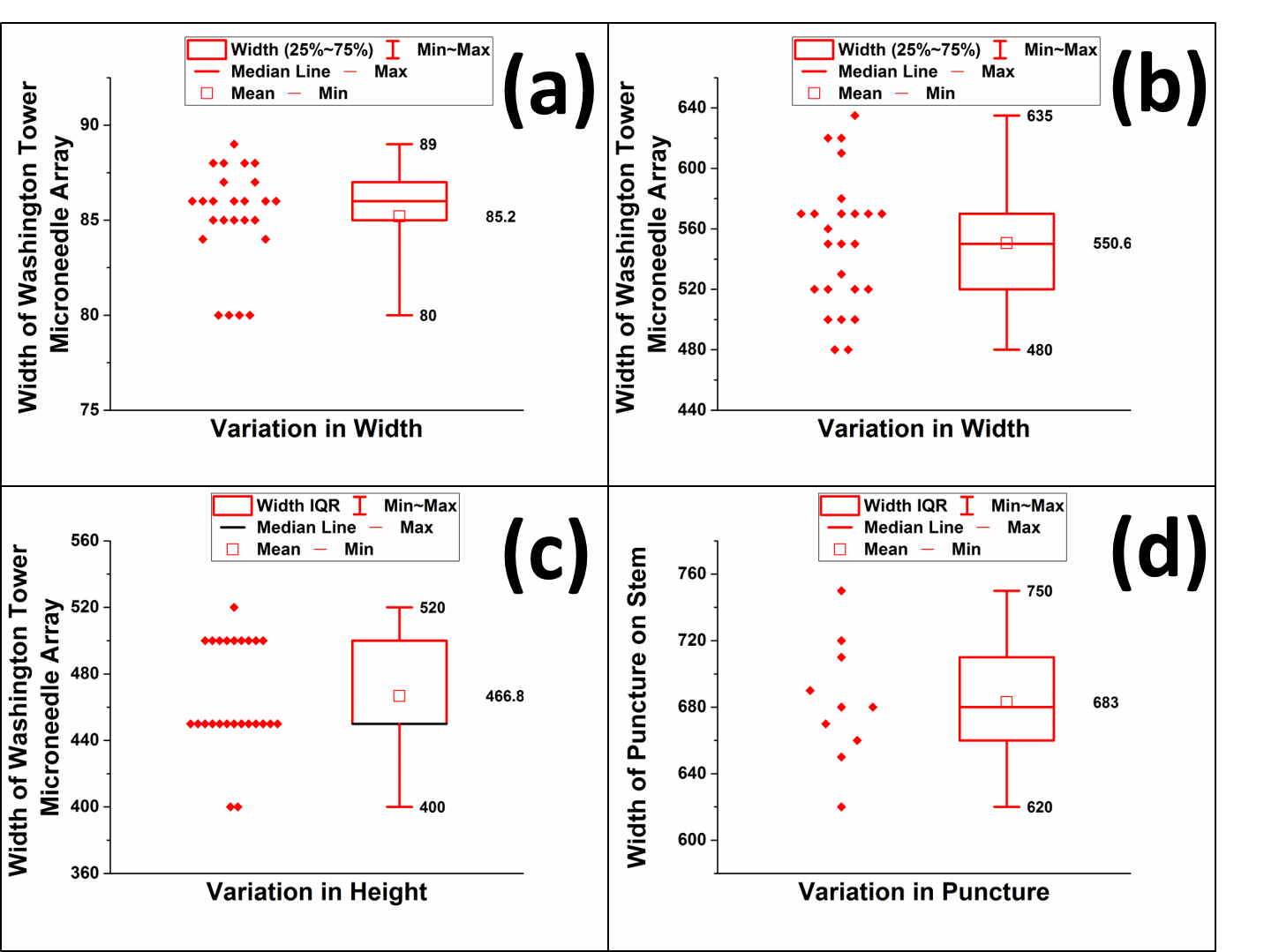


Figure S1: (a) Tight angular distribution (ϴ) of 85.2º with the horizontal which shows the efficiency of the Hypo-Rig in transitioning the micro and *mesoneedles* out of plane. A box plot of N=25 µMMNs showing variation in base (b) width and (c) height after micromilling. (d) A box plot of N=10 puncture sites showing variation in puncture width.

**
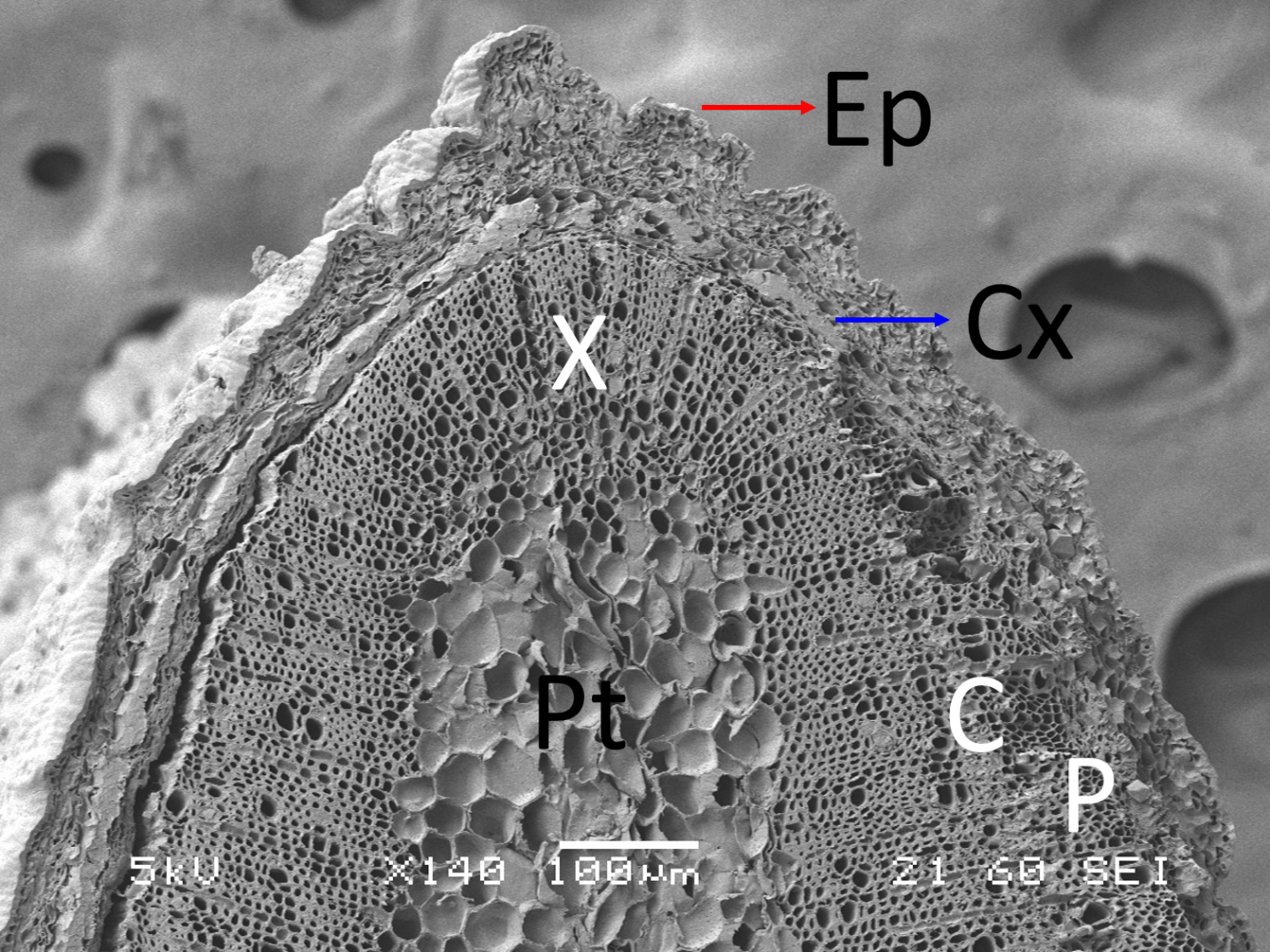
**

Figure S2: SEM image showing the cross-section of the un-punctured stem with the epidermis (Ep), cortex (Cx), phloem (P), cambium (C), xylem (X) and the pith (Pt)


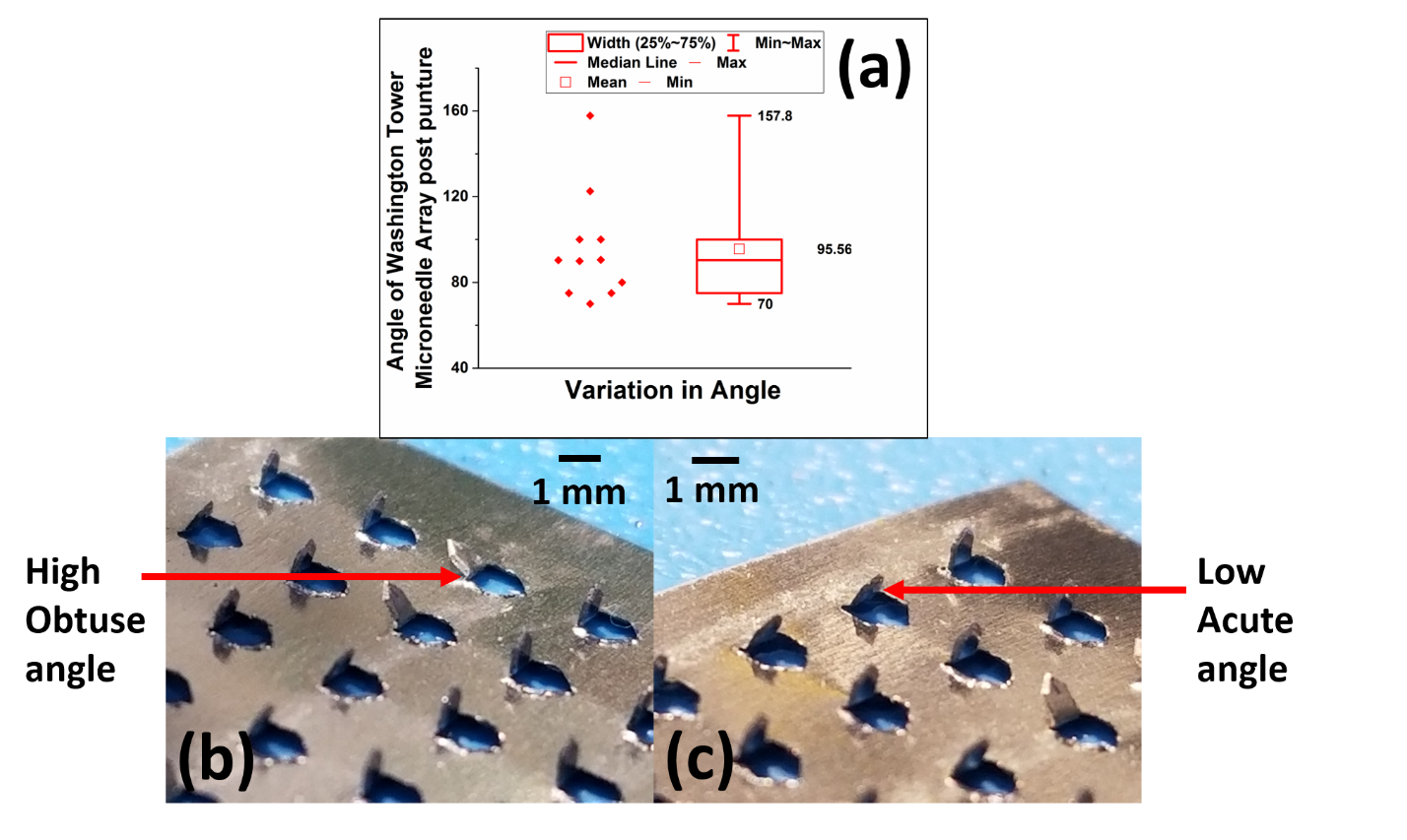
Figure S3: (a) Box plot of N=10 µMMNs which have been bent from their original positon after pressing onto the stem surface. Representative images of µMMNs dislocated to (b) higher values of obtuse angles or (c) low values of acute angles after pressing into plant stem.

**
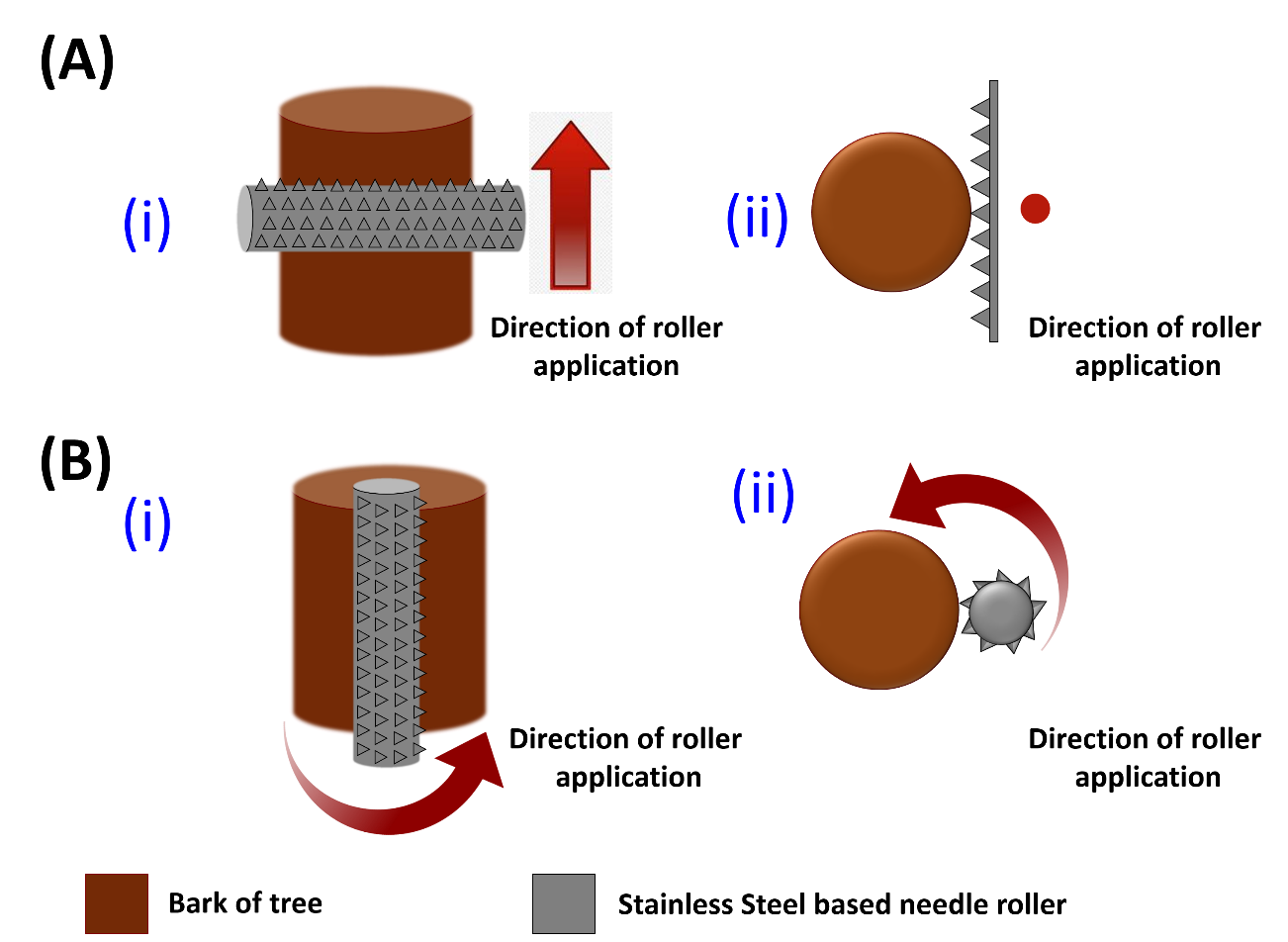
**

Figure S4: (A) (i) 3D Schematic of the needle roller being applied vertically, (ii) top-view showing the axis of the roller based needle array being a tangent to the axis of the cylindrical tree trunk while moving into the plane. (B) (i) 3D Schematic of the needle roller being applied horizontally, (ii) top-view showing the roller needle array engaging with the full surface of the tree trunk as it is being rolled.
